# The Development of Single-Cell Metabolism and Its Role in Studying Cancer Emergent Properties

**DOI:** 10.3389/fonc.2021.814085

**Published:** 2022-01-10

**Authors:** Dingju Wei, Meng Xu, Zhihua Wang, Jingjing Tong

**Affiliations:** ^1^ School of Life Science, Central China Normal University, Wuhan, China; ^2^ Shenzhen Key Laboratory of Cardiovascular Disease, Fuwai Hospital Chinese Academy of Medical Sciences, Shenzhen, China; ^3^ State Key Laboratory of Cardiovascular Disease, Fuwai Hospital, National Center for Cardiovascular Disease, Chinese Academy of Medical Sciences and Peking Union Medical College, Beijing, China

**Keywords:** cancer metabolism, metabolic reprogramming, metabolic heterogeneity, single-cell metabolomics, tumor drug resistance

## Abstract

Metabolic reprogramming is one of the hallmarks of malignant tumors, which provides energy and material basis for tumor rapid proliferation, immune escape, as well as extensive invasion and metastasis. Blocking the energy and material supply of tumor cells is one of the strategies to treat tumor, however tumor cell metabolic heterogeneity prevents metabolic-based anti-cancer treatment. Therefore, searching for the key metabolic factors that regulate cell cancerous change and tumor recurrence has become a major challenge. Emerging technology––single-cell metabolomics is different from the traditional metabolomics that obtains average information of a group of cells. Single-cell metabolomics identifies the metabolites of single cells in different states by mass spectrometry, and captures the molecular biological information of the energy and substances synthesized in single cells, which provides more detailed information for tumor treatment metabolic target screening. This review will combine the current research status of tumor cell metabolism with the advantages of single-cell metabolomics technology, and explore the role of single-cell sequencing technology in searching key factors regulating tumor metabolism. The addition of single-cell technology will accelerate the development of metabolism-based anti-cancer strategies, which may greatly improve the prognostic survival rate of cancer patients.

## Background

Cancer is one of the top lethal factors, and cancer patients bear a heavy burden of life expectancy globally. According to the latest data reported by World Health Organization’s International Agency for Research on Cancer (IARC), up to 19.3 million new cancer cases and approximately 10.0 million cancer deaths occurred in 2020 worldwide (1). Therefore, the development of early diagnosis and effective treatment of cancer is urgent.

One of the most significant features of cancer is metabolic reprogramming, and increasing evidence suggests that dysregulated cell metabolite facilitates tumor initiation, progression, metastasis, and drug resistance. In 1924, Otto Warburg firstly identified that cancer utilizes glycolysis instead of oxidative tricarboxylic acid cycle (TCA) to provide energy, nucleotide, lipid, and amino acid for the growth even under aerobic conditions (2). Later in 1975, Ambanelli reported that malignant tumors exhibited congenital errors of the degradation of tryptophan (3). Several years later, it was found that glutamine is the most rapidly consumed amino acid in proliferating Ehrlich ascites carcinomas and also a number of hepatomas and carcinosarcomas (4). Therefore, understanding metabolic alterations in cancer cells may give us a hint to discover new therapeutic targets and facilitate oncology drug research and development of cancer therapy. The idea of cancer metabolism-based therapy has been raised for decades, but significant side effects of antimetabolite drugs, which are caused by destroying normal rapidly proliferating cells, made them limited in preclinical studies (5). For instance, although numerous preclinical studies have manifested the anti-proliferative effects of 2-deoxyglucose (2-DG) (6), the usage of 2-DG was limited by its toxicity related to hypoglycemia symptoms (7). Recent clinical trials have illustrated that lower doses of 2-DG are insufficient to inhibit disease progression (8, 9). Moreover, although lactate dehydrogenase-A (LDH-A) was frequently identified as an overexpression gene in human cancers (10), none of the LDH-A inhibitors have reached clinical trials as effective antimetabolite chemotherapy drugs, suggesting either insufficient drug exposure, unacceptable toxicity (11), or a lack of LDH-A dependence in human tumors. Similarly, two diabetes therapeutic biguanide compounds metformin and phenformin have been found to reduce tumor growth (12), however, in cells lacking a functional LKB1 pathway, the biguanide drugs have been demonstrated to result in rapid apoptosis (13).

**Figure 1:**
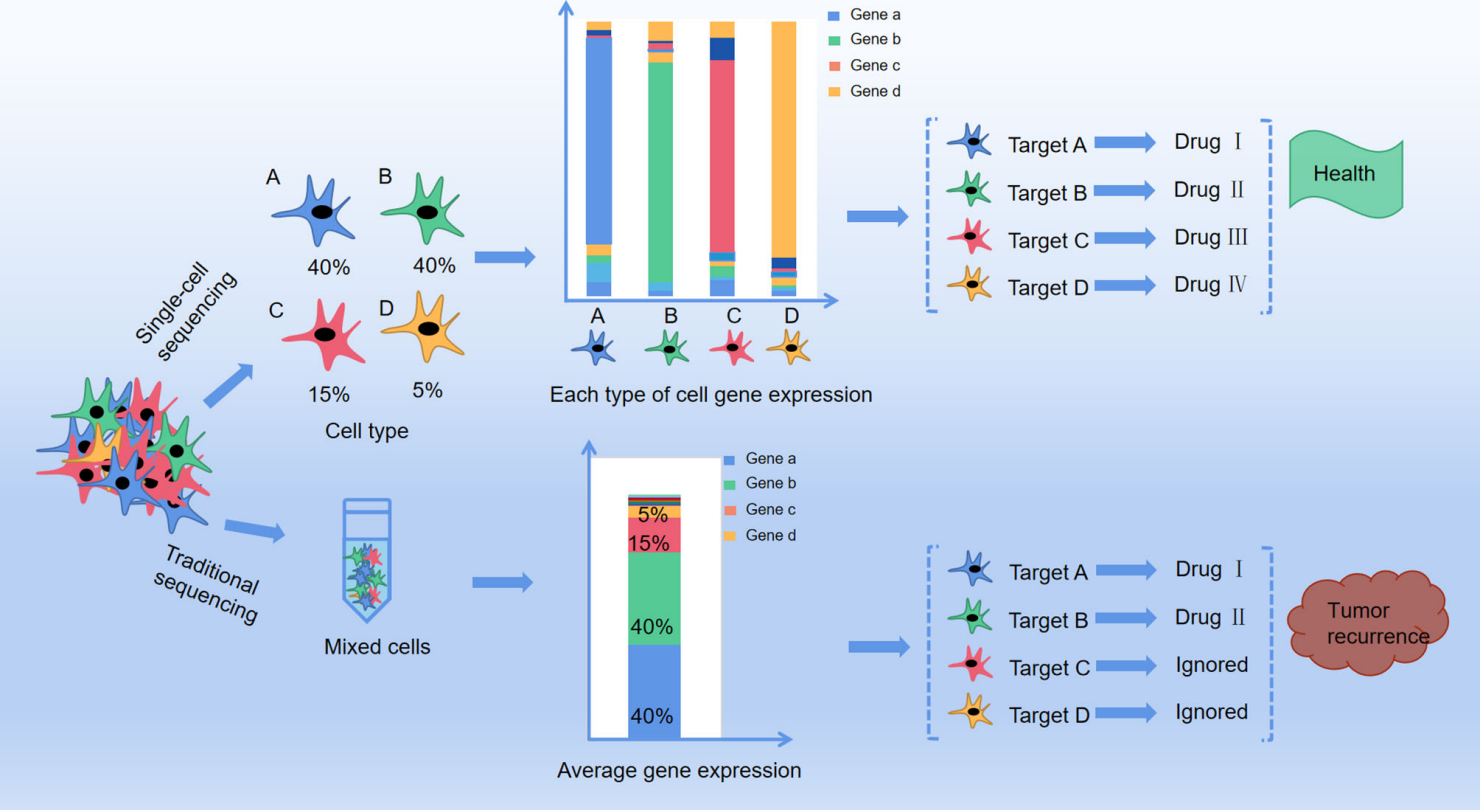
The difference between traditional method and single cell method in profiling anti-cancer targets. A、B、C and D represent four different cell types in cancer tissue with different distribution (A: 40%, B:40%, C:15%, D:5%), and each type of the cells present a target gene as gene a, b, c and d respectively by single cell sequencing. Drug I, II, III and IV are targeted drugs for gene a, b, c and d respectively, and with co-administration of drug I, II, III and IV the cancer is completely curable. However, with traditional RNA-sequencing, mixture of cell A, B, C, and D only presented target gene a and b With co-administration of drug I and II, only group of cell A and B are killed but cell C and D are still survival, leaving a chance for tumor recurrence.

In this scenario, would interfering cancer cell metabolism be wrong? Looking back at previous studies, we found that on the one hand, since multiple metabolic pathways altered in tumor cell, by only interfering one metabolite is not sufficient for cancer treatment. On the other hand, some metabolic targets of cancer are also essential for the normal cells growth, so it is very important to control the dosage of tumor metabolism therapeutic drugs. In addition, considering the traditional metabolomics analysis method, the proposed therapeutic targets have been extracted from the average information of a cell mixture. In reality, recent research has confirmed that even from the same tissue, individuals of the same group of cells are different because of cell heterogeneity. Cell heterogeneity is particularly common in tumor tissues, as the fact that some specific cells survived by altering their metabolism after chemotherapy, therefore cancer may re-emerge years later (14, 15). And single-cell analysis can identify the intracellular biochemical components and features, and at the same time can study the relationship between metabolism and cell function, cell development, and differentiation. Using this method, the pathological mechanism of cells in disease states can be studied, and it is also helpful for clinical diagnosis and prognosis (16, 17) (**Figure 1**). In addition, this technology can also understand detailed cell information and reduce the problems caused by statistics and data processing in previous research methods.

In this paper, we summarized the research progression of single-cell metabolomics analysis technology, mainly from two aspects: (1) Research status of single cell metabolomics technology; and (2) Application of single cell metabolomics in tumor research.

## The Development of Single-Cell Metabolomics Techniques

**Figure 2:**
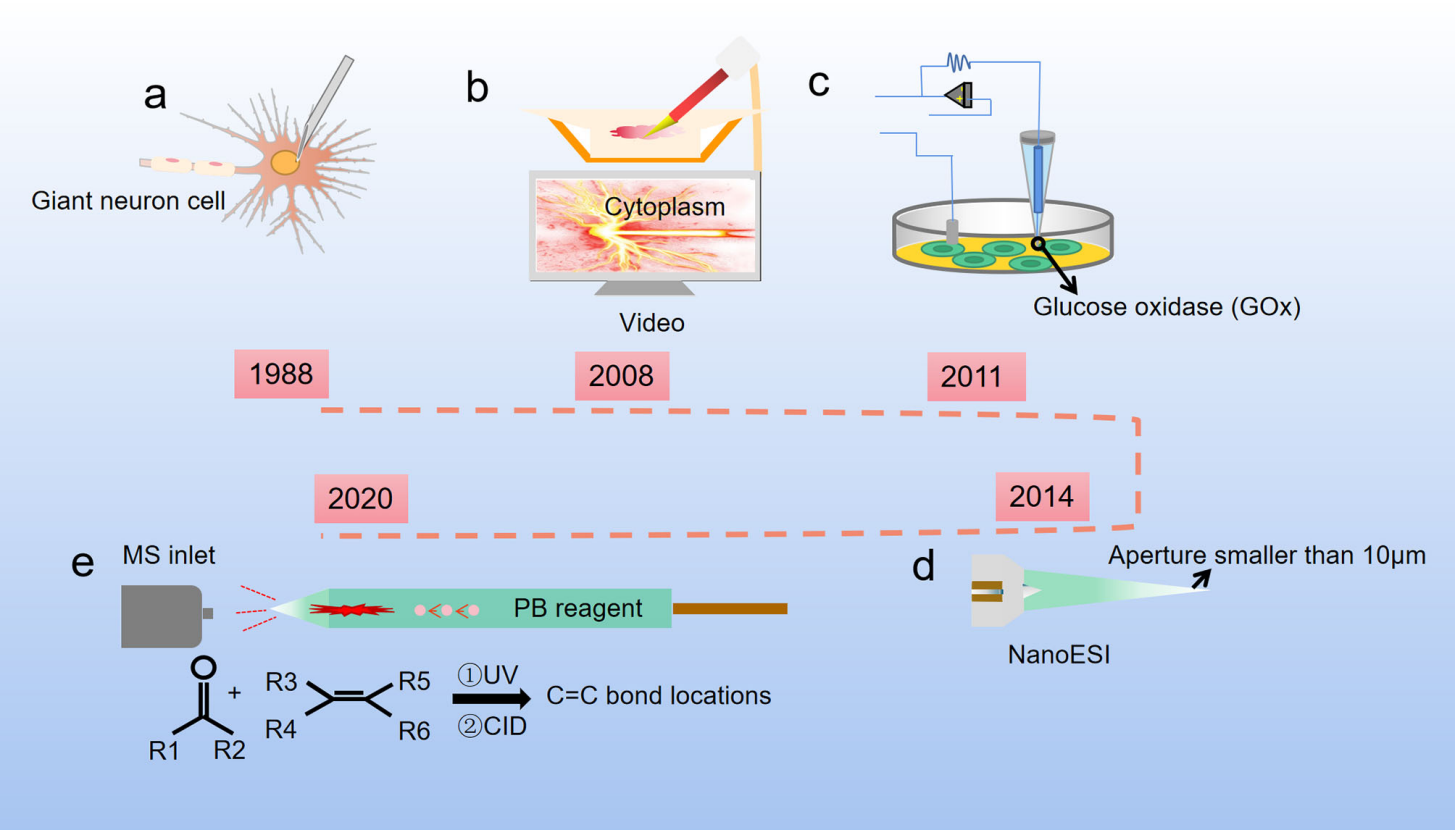
Development of single cell metabolite extraction. **(A)** Microsyringes composed of glass straws were firstly used to extract metabolites from giant neuron cells of snails (1988); **(B)** Nanospray ionization captures the sample from a single living cell, and this process is controlled by a cell manipulation system (2008); **(C)** A nanotube that can measure glucose levels in single-cell level (2011); **(D)** The Single-probe coupled with a sampling tip size < 10 μm, is a miniaturized multifunctional sampling and ionization tool which can achieve *in situ* metabolomic analysis of individual living cells with real-time performance (2014); **(E)** The micropipette needle accommodated with PB reactions inside to determine C=C bond locations in unsaturated lipids at single-cell level (2020).

The first single-cell metabolomics was established by Kennedy and Jorgenson. They used open tubular capillary chromatography to analyze the amino acid composition of a snail single giant neuron (**Figure 2A**) (18, 19). At the same time, Wallingford and Ewing reported sampling the internal contents of a single giant neuron by using a capillary (20). However, considering the different size and volume between the giant neurons and normal cells, the method for extraction of single cell metabolites needs to be suitable for different cell sizes (21). Moreover, the metabolic pathways in cells can be easily affected by both internal and external factors and are highly dynamic, which is very different from the genome, transcriptome, and proteome. Compared with single-cell genomics, transcriptomics, and proteomics, single-cell metabolomics can provide the most sensitive dynamic picture for understanding cell functions, but the measurement of single-cell metabolomics targets is undoubtedly the most difficult. One of the main problems in the preparation for single-cell samples is how to avoid or reduce the impact on cell metabolism during the sample preparation process. In addition, the content of substances in single cells is low, which puts forward higher requirements for the sensitivity of the detection method. Finally, there are many kinds of metabolites in a single cell, and the concentration difference of metabolites can be as high as 10^6^~10^9^ times. This requires the detection method not only to respond to multiple substances at the same time, but also to have a wide response range. In short, single-cell analysis technology requires high sensitivity, small sample size, good selectivity, fast response speed, and no impact on cell status, while the data analysis requires complex techniques and models.

### The Single Cell Metabolite Extraction

Cell sampling is the first and arguably the most critical step in single cell analysis. To avoid or reduce the impact of environmental changes on cell metabolism during the sample preparation process, one of the methods is to maintain cells in a natural environment as much as possible. For example, use microfluidic chips for cell culturing after cell separation, inject quantitative chemical substances into the culture medium, and selectively release the cells for analysis (22, 23). Another method is to perform rapid freezing of the cells before the metabolite determination to prevent the cells from undergoing dramatic changes in the metabolites (24). The extraction tools for single cell metabolites have been optimized over the past decades. Hajime Mizuno successfully established a direct and rapid analysis of the locations and the metabolic pathways of tryptophan and histidine metabolites in a live rat basophil leukemia cell by live single-cell video-mass spectrometry. The contents of the cell were sucked into a nano-electrospray ionization (nano-ESI) tip, dissolved in an ionization solvent, and directly introduced into a quadrupole-time of flight mass spectrometry (Q-TOF-MS) by nano-spray (**Figure 2B**) (25). Later, based on Mizuno’s research, Ning Pan developed a new miniaturized multifunctional sampling and ionization device, the Single-probe, with a sampling tip size smaller than 10 μm, which can be inserted into single cells to extract intracellular compounds. Multiple endogenous and exogenous cellular metabolites in a single living eukaryotic cell could be analyzed in real-time by directly coupling the new probe with the mass spectrometer (**Figure 2D**) (26, 27). Single cell mass spectrometry (SCMS) enables to obtain higher sensitivity and accuracy of chemical information at the single-cell level, which could improve our understanding of biological and pharmaceutical bioanalytical research compared with previous methods.

Raphael proposed a different method for metabolite extraction, he combined glucose oxidase (GOx) covalently with the nanopipettes tip (**Figure 2C**), and the tip was functionalized as glucose nanosensors to quantify single cell intracellular glucose levels. During and after the nanopipette measurement, the cells remain viable. Therefore, nanopipette-based glucose sensors provide a way to compare changes in glucose levels with changes in cell proliferation or metastasis. The nanopipette-based glucose sensors has broad prospects as a diagnostic tool for distinguishing cancer cells from nonmalignant cells in heterogeneous tissue biopsies and a tool for monitoring cancer progression *in situ* (28). Later, Yanlin Zhu has developed a new technology that uses a silica capillary fused micropipette needle, in which can induce Paternò-Büchi (PB) reactions at the C═C bond, and locations of C═C bonds in unsaturated lipids can be determined in cell lysate at the single-cell level (**Figure 2E**). The capillary needle exhibits multiple functions including single cell metabolite extraction probe, cell lysis container, micro-reactor, and nano-ESI emitter during the measurement of metabolites in a single human colon cancer cell HCT-116. This technique is potentially able to apply in other reactive SCMS studies to enhance molecular analysis for broad ranges of single cell metabolites (29).

In addition, mitochondria are important organelles where glucose metabolism happens. Studies on extraction of metabolites in mitochondria are also proposed. Tsuyoshi Esaki combined fluorescence probing with live single-cell mass spectrometry, directly analyzed of mitochondria metabolism in a live HepG2 cell. They stained mitochondria of target cells by fluorescence probe and directly sucked the mitochondria into the nanospray tip under a micromanipulator operation. The sample was then sent to a high-resolution mass spectrometer LTQ-Orbitrap Velos Pro equipped with a nano-electrospray ionization source, and the final result was analyzed by comparing the data gained from stained mitochondria with unstained cytosol blank samples. This fluorescence imaging technique opens the door to analysis of site- and state-specific molecular detection to clarify the precise molecular principles at the level of single-cell and organelle (30).

### The Single-Cell Metabolite Flux Detection

Researchers usually use mass spectrometry (MS) or nuclear magnetic resonance (NMR) to carry out metabolomics research, but because NMR technology is not very sensitive, MS has become the main method. However, single-cell analysis is still a tough challenge even with recent technologies, because unlike genes, metabolites cannot be amplified. To analyze such a tiny amount of metabolites in a single cell, many efforts have been tried to improve both detection sensitivity and ionization techniques in MS. On the one hand, considering the small amount and wide dynamic concentration ranges of metabolites in an individual cell, researchers have considered amplifying the signal of the small amount of analyte. Richard B. Keithley administrated three fluorescently labeled glycosphingolipid substrates, GM3-BODIPY-FL, GM1-BODIPY-TMR, and lactosylceramide-BODIPY-650/665 to simultaneous probe metabolism at three different points in the cascade of glycolipid metabolism in HCT 116 spheroids. Finally, they found cells from different regions of HCT 116 spheroids exhibited differences in metabolism, and this three-color fluorescence labeling dramatically amplified the signal of metabolites (31). On the other hand, the direct injection of single cells separated by microfluidic devices or micropipettes into MS suggested the possibility of highly sensitive metabolite analysis in single cells. Hsiao-Wei Liao applied field amplified sample injection (FASI) to capillary electrophoresis electro spray ionization mass spectrometry (CE-ESI-MS) to detect intracellular metabolites from a single neuron, and achieved 100- to 300-fold enhancement of detection limit compared to normal injections. The analytes identification and quantification accuracy were further enhanced through the introduction of internal standards (32). Expect for amplifying the signal of the detected substance, to increase sensitivity of the detection equipment can also achieve the same goal. Takayuki Kawai firstly developed “nano-CESI” emitter, which has a thin conductive wall (10μm) and tapered (5-10μm) end. Compared with a conventional sheathless emitter, the nano-CESI emitter provided up to 3.5-fold increase in sensitivity, and by coupling a sample enrichment method, large-volume dual preconcentration by isotachophoresis and stacking (LDIS), up to 800-fold increase of sensitivity has been achieved compared with normal sheathless CE-MS in total (33).

Mass spectrometry imaging (MSI) is a powerful tool that advances our understanding of complex biological processes by revealing unprecedented details of metabolic biology. MSI does not require labeling, so it can analyze any compound present on the tissue, which is in stark contrast to most label-based imaging methods, which require prior knowledge of clearly defined targets. In addition, hundreds of compounds can be imaged simultaneously in MSI, which is different from traditional optical imaging in which by using limited number of different fluorescent label colors, only a few targets can be imaged at a time. MSI can be performed by combining any desorption ionization technique with MS in microprobe mode, with the application of matrix-assisted laser desorption ionization (MALDI), the laser can be focused to a micrometer size, thereby reducing the sampling area to a subcellular size. By using the high spatial resolution MALDI-MSI, high-precision metabolite positioning can be obtained at cellular and subcellular levels *in situ* (34).

### Single-Cell Metabolomics Data Analysis

In addition to optimize the methods of sample preparation and detection, high-throughput information acquisition is also essential in single cell metabolomics analysis to understand the metabolic process in a single cell. Although the application both of traditional bioinformatics methods and unspecialized software MassLynx (35, 36) to interpret the experimental results of single-cell metabolomics is not a problem, the main challenge is to develop a method that can analyze multiple single cells at the same time and detect the metabolites in each cell while making the results statistically significant. Renmeng Liu analyzed combined SCMS experiments with a generalized integrated data analysis workflow, including data preprocessing, visualization, statistical analysis, machine learning, and pathway enrichment analysis, to conduct single cell metabolomics studies of live cancer cells to discover phenotypic biomarkers and unveil related biological pathways changes during liver cancer chemotherapy (37). Luca Rappez applied SpaceM together with a fluorescence-based readout to detect >100 metabolites from >1,000 individual cells per hour with retention of morpho-spatial features (38). To minimize labor-intensity and enhance the analytical sensitivity, Anqi Chen combined mass spectrometry analyses with a visual serving robotic micromanipulation platform, which sequentially extracted, aspirated, and ionized single cells. This system is the first automated single cell mass spectrometry (SCMS) system. Compared with traditional methods, the automated SCMS system functions without manual operation and facilitates a high-performance single cell metabolic analysis (39).

### Chapter Summary

**Figure 3:**
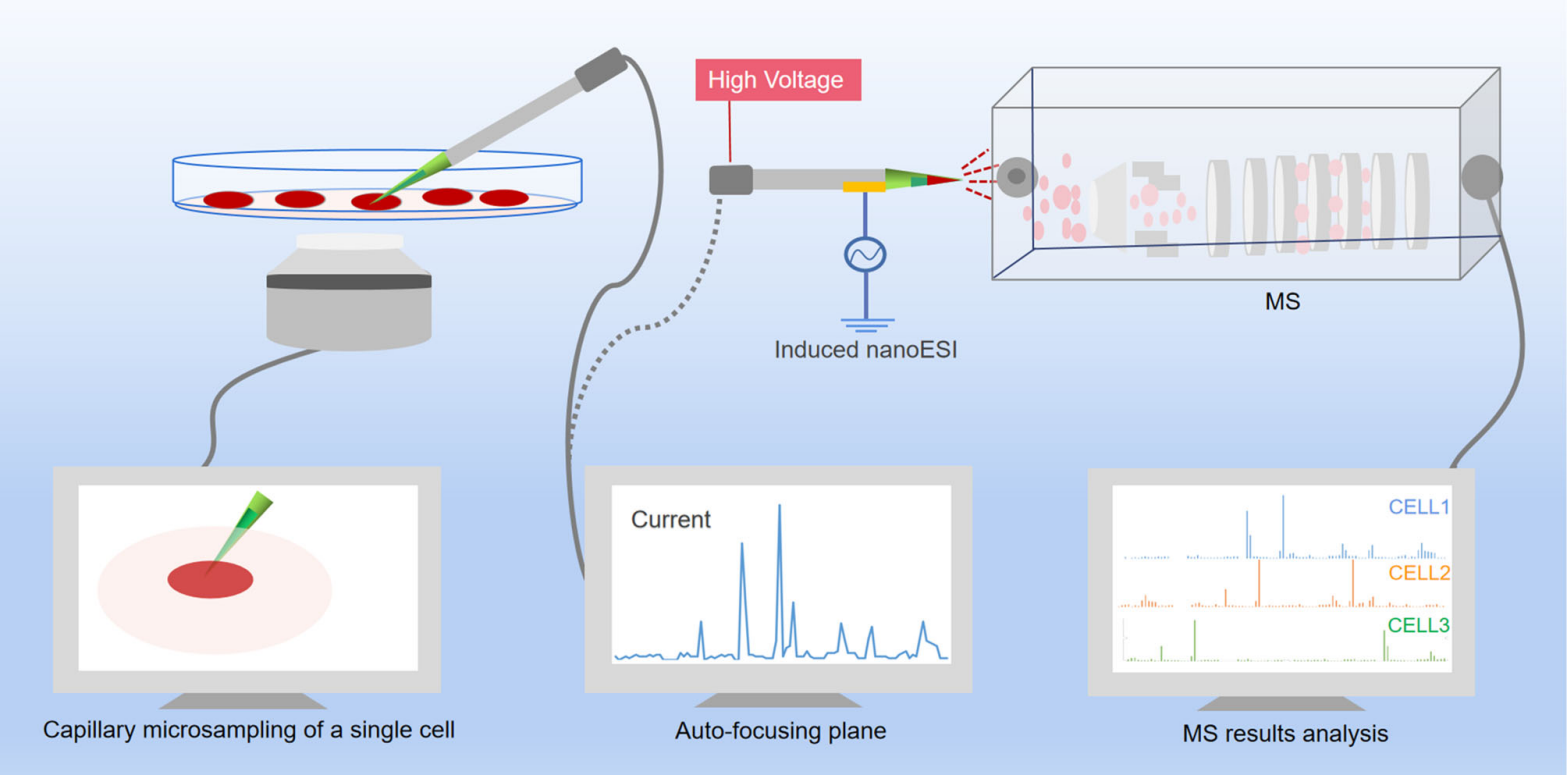
Scheme of single-cell metabolomics analysis of live cell. The content of a live single cell is extracted by micropipette needle under the guidance of an inverted micro-scope, and a cell manipulation system was used to control the micropipette needle to aim the targeted cell. After assistant solvent was added to the sampling tip, the biomolecules were dissolved into the assistant solvent and immediately ejected from the tip into the MS under the impetus of the electric field. The raw data then analyzed by software.

The typical analysis of living cells is a delicate process, mainly start with using nano ESI capillary to extract the contents from the cells, and then use the same capillary to inject the contents directly into the MS. The conceptual schematic diagram of the workflow of MS analysis of single cell metabolites is shown in **Figure 3**. However, the current single-cell metabolomics technology is still under the way to get more refined. Extraction, detection and analysis are the three major difficult problems which must be resolved. With continuous optimization of current single-cell metabolomics techniques, a comprehensive portrait of metabolic features of each unique cell can be expected in the near future.

## The Application of Single-Cell Metabolomics in Cancer Research

The detection and understanding of cancer cells is one of the most important the potential applications of single-cell metabolomics. One application is the discovery of cancer cells with abnormally high metabolic rates within cells undergoing normal metabolism, including circulating tumor cells that cause cancer metastasis (40, 41). In current cancer treatment, single-cell metabolomics technology can be applied to dig out why some cancer cells are still able to survive after being stimulated by the environment or drugs by altering their metabolic pathways (42, 43). Other potential applications single-cell metabolomics technology include understanding the mechanism of tumor metastasis (44, 45) and obtaining the input and output data required to establish a mathematical model of cell metabolism and to learn more about the fate of cancer cells (46).

### Single-Cell Metabolomics Reveals Mechanisms of Tumor Drug Resistance

During tumor chemotherapy, part of the patients develop drug resistance, resulting in treatment failure and tumor recurrence, which causes more than 90% of cancer-related deaths. Solid cancers have shown the intratumor heterogeneity spatially and temporally, which has become the biggest obstacle in tumor therapy (47, 48). Single-cell technology is a powerful tool to analyze tumor heterogeneity, and it pulls the dimension of our observation of tumors to the dimension of a single cell (49). By analyzing the genetic and metabolite information of individual cells, we can distinguish genes and regulatory pathways driving drug resistance development (50). For example, Renmeng Liu exposed HCT-116 cells to taxol and vinblastine, which are two mitotic inhibitors, under a series of treatment conditions, then they used Single-probe SCMS system to measure metabolomics change in cells. Phenotypic biomarkers related to the emerging phenotypes resulted from drug treatment were discovered and compared through a series of rigorous statistical analysis with the single cell study and traditional liquid chromatography-MS (LC-MS) study from bulk cell samples. Through pathway enrichment analysis, four biological pathways that may be involved in the drug treatment of colorectal carcinoma have been identified, and this technique can be potentially applied to future pharmaceutical and chemotherapeutic research (51). Clinical studies demonstrated that high concentration (10 μM) erlotinib inhibited cancer proliferation, but beyond the normal tolerance level, while low concentration (1 μM) erlotinib exhibited no treatment effect. Based on Xue Min’s research, the low-dose (1 μM) of erlotinib actually increased the energy potential of cells, even if glucose uptake and phosphoprotein signaling were inhibited, which may help explain the resistance of some cancer patients to EGFR inhibitors (52). Mei Sun used the Single-probe mass spectrometry (MS) technique to inspect the metabolic features of individual live colorectal cancer stem cells (CSCs). Comparison with non-stem cancer cells (NSCCs), she/he found CSCs contained relatively higher amount of tricarboxylic acid (TCA) cycle metabolites and unsaturated lipids. Application of inhibitors of stearoyl-CoA desaturase-1 (SCD1), nuclear factor κB (NF-κB), and aldehyde dehydrogenases (ALDH1A1) in CSCs significantly contracted the abundance of unsaturated lipids and hindered the formation of tumor spheroids, leading to reduced stemness of CSCs. This indicates that single-cell metabolomics can potentially be used for metabolomics research on rare types of cells, and provides a new method to discover functional biomarkers as therapeutic targets (53). Yapeng Su integrated single-cell flow cytometry with theoretical investigation to study the cell-state transition dynamics associated with BRAF inhibitor drug resistance in BRAF-mutant melanoma cell. They concluded that in certain plastic cancers, the population heterogeneity and evolution of cell phenotypes may be comprehended by explaining the competitive interaction between the epigenetic potential landscape and state-dependent cell proliferation. Their research suggested that experimentally verifiable predictions can potentially determine the trajectories that single BRAFV600E mutant melanoma cancer cells take between drug-naive and drug-tolerant states and guide the design of effective treatment strategies (54). Therefore, the resolved heterogeneous drug-response trajectories by single-cell technique update our current understanding of how drug resistance developed and can provide a powerful methodology for identifying effective combined treatment (55).

### Single-Cell Metabolomics Reveals Tumor Metastasis

Much effort had also been made to explore the predictive genomic changes in disease prognosis (56, 57). Recent studies showed that even individual cells from the same clonal can display a broad landscape of different properties, such as different patterns of gene expression (58) and invasive behaviors (59), further increased the challenge of deciphering the mechanism of metastasis in cancer. In this scenario, single-cell omics are applied in tumor metastasis studies. For instance, Ryan T. Davis used single-cell RNA sequencing, flow cytometric and metabolomics to analyze patient-derived-xenograft models of breast cancer, and they found breast cancer micrometastases display a distinct metabolic profile and many of them implicated with metastasis—such as glutamine, fatty-acid and proline metabolism. Most importantly, they found the breast cancer micrometastases converged on or produced critical metabolites to drive oxidative phosphorylation (OXPHOS), and pharmacological inhibition (oligomycin) of OXPHOS substantially attenuates lung metastasis (60). Circulating tumor cells (CTCs) which are released from primary tumor lesion sites into the blood circulation are an important source of tumor metastasis to distant body organs (61). Yasmine Abouleila analyzed untargeted molecular profile of single CTCs collected from gastric cancer (GC) and colorectal cancer (CRC) patients by using live single cell mass spectrometry integrated with microfluidics-based cell enrichment techniques. The authors revealed significant differences in metabolites between the CTCs and lymphocytes from the same patient, and Principal component analysis-discriminant analysis (PCA-DA) showed obviously different clustering behavior between CTCs and lymphocytes in each cancer. And due to the metabolic differences between GC and CRC, CTCs were clustered into two different groups corresponding to different cancer types, suggesting that the characteristics of CTCs metabolome may become a tool for cancer diagnosis in the future (62). According to the above studies, we learn that at single cell level, it is possible to find new potential biomarkers which promoting tumor metastasis and accelerating tumor deterioration that cannot be detected by traditional methods. In addition, different types of cancer can be classified based on their metabolic fingerprints, which may play a role in identifying clinical targets that may slow down or prevent tumor metastasis.

### Single-Cell Metabolomics Reveals Cell Fate

**Figure 4:**
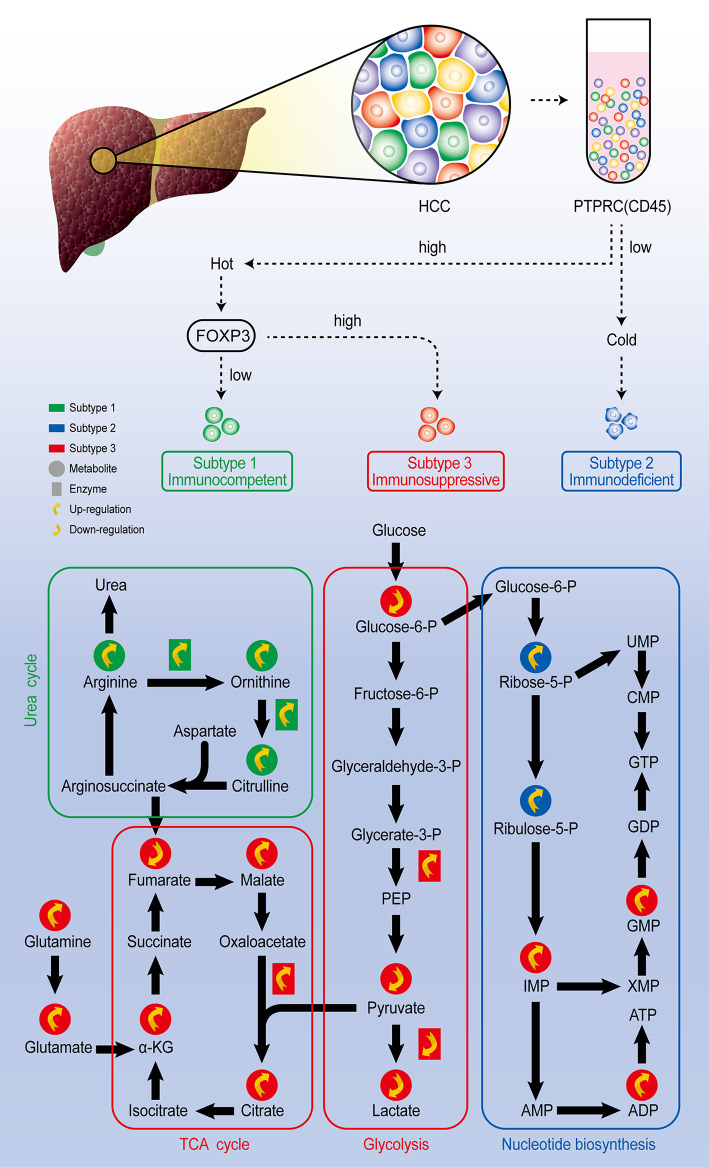
Comprehensive exploration of the heterogeneity of HCC analysis scheme. Through single-cell sequencing, HCC could be classified as three subtypes according to the expression level of marker genes PTPRC and FOXP3. Further single-cell metabolomic analysis revealed that the immunocompetent subtype is with upregulated urea cycle, the immunosuppressive subtype is with upregulated TCA cycle and inhibited glycolysis pathway, and the immunodeficient subtype presents upregulated nucleotide biosynthesis pathway.

Single-cell technology also allows you to track the state change of the cells under different environment. Qi Zhang published a comprehensive study on the heterogeneity of Hepatocellular carcinoma (HCC) from genome to phenotype and from single-cell level to body level. By single cell sequencing, they classified HCC as the immunocompetent subtype, immunodeficient subtype, and immunosuppressive subtype, respectively. Among three subtypes of cells, the immunosuppressive subtype showed inhibited glycolysis and enhanced mitochondrial respiration, the immunodeficient subtype showed increased nucleotide biosynthesis, while immunocompetent subtype was featured by upregulated urea cycle (**Figure 4**). They also found that although the heterogeneity of tumor cells is significant in all dimensions, the local immune status of HCC is less heterogeneous, therefore they believe that targeting local immunity might be suitable for HCC treatment (63). To track the state of cells, Felix J. Hartmann employed single-cell metabolic regulome profiling (scMEP), an approach applies antibody-based assays to analyze cellular identity and metabolic regulation in the single-cell level. They used a mass spectrometry flow cytometry, time-of-flight flow cytometry (CyTOF), to compare scMEP with a large number of metabolic assays by reconstructing the metabolic remodeling of naive and memory CD8+ T cells activated *in vitro*. Based on the changes in the expression of metabolic characteristics over pseudo-time, three inflection points in the metabolic remodeling process of naive human CD8+ T cells were defined. The first inflection point was marked by the concerted and accelerated up-regulation of metabolic proteins, such as GLUT1, ASCT2, OGDH and VDAC1, leading to the second inflection point, which was characterized by the onset of RNA synthesis and activated cellular stress responses. The third metabolic inflection point was defined by the expression levels of stable or reduced metabolic proteins, such as GLUT1 and ASCT2, and peak translation activity. Therefore, the application of scMEP allows us get a better understanding of tumor–immune boundary and helps identify disease-related metabolic changes, which can be used as potential biomarkers and therapeutic targets for various human diseases (64).

If the cell identity can indeed exist as a continuum, this provides an opportunity to stabilize the transient phenotype and create new cell identities, giving new functions to known cell types. These questions are also related to the field of cell fate reprogramming. At least, we will obtain a high-resolution template to summarize the identities of the main functional cell types. Through the above analysis, we fully understand that single-cell metabolomics techniques serve as a fast, high-throughput predictive tool in cancer research, which can predict metabolite targets in cancer therapy and pharmacology. At the same time, single cell metabolomics is expected to find reliable and effective clinical biomarkers for cancer prognosis and diagnosis.

## Conclusions

In the past decade, single-cell technology has pushed biological research into a new era of exploring cellular and molecular phenotypes at an unprecedented level of resolution. This progress is mainly due to the innovative progress of high-throughput technology and the development of new computing tools, which enable us to capture the genome, transcriptome, proteome, metabolic status, and other multi-dimensional information of thousands of single cells at the same time without relevant information. With these emerging technologies, we can study cell types, cell states, and individual cell responses to external stimuli or internal biological processes. Although metabolomics started late, with the continuous advancement and innovation of detection technology, more breakthroughs will surely be made in the future. Nevertheless, before single-cell metabolomics is truly applied to systems biology and medical diagnosis of cancer research, some challenges of experimental technology or bioinformatics still need to be overcome. There are still many problems need to be solved, including expanding the coverage of metabolites in living cells, faster identification of single-cell metabolites while achieving high-throughput detection, and reduce cost.

In the next few years, greater progress is likely to be made toward new combinations of different single-cell omic techniques to capture of all molecules in an individual cell. We also expect that by further combining existing indexing or microfluidic technologies, technologies that can analyze four or five-layer omics data in parallel can be developed, resulting in single-cell multiomics methods capable of high-throughput processing of thousands of cells. Currently, each omics data set need to be analyzed separately and compare the final results to get the conclusion, therefore novel computational methods and specialist multiomics algorithms that allow the integrated study of two or more omic layers per cell of large heterogeneous populations and data analysis of the different layers together will also need to be developed. Future multi-omics technologies will eventually need to cover all single-cell omics technologies in order to characterize these omnisciently for different layers, three-dimensional coordinates, phenotypes, and cell lineage history.

## Author Contributions

JT, DW, and MX conceived and designed the review. DW did paper search and DW and MX wrote the manuscript. JT had supervised this project and contributed to writing and revision of the manuscript. All authors contributed to the article and approved the submitted version.

## Funding

This study was supported by grants from the National Natural Science Foundation of China (NSFC No. 32170763), the National Natural Science Foundation of Hubei (ZRMS2020002147) and the Research Funds from Central China Normal University (CCNU20TS017).

## Conflict of Interest

The authors declare that the research was conducted in the absence of any commercial or financial relationships that could be construed as a potential conflict of interest.

## Publisher’s Note

All claims expressed in this article are solely those of the authors and do not necessarily represent those of their affiliated organizations, or those of the publisher, the editors and the reviewers. Any product that may be evaluated in this article, or claim that may be made by its manufacturer, is not guaranteed or endorsed by the publisher.
